# Perceptions of and willingness to engage in public health precautions to prevent 2009 H1N1 influenza transmission

**DOI:** 10.1186/1471-2458-11-152

**Published:** 2011-03-08

**Authors:** Marc T Kiviniemi, Pavani K Ram, Lynn T Kozlowski, Kaitlin M Smith

**Affiliations:** 1 School of Public Health and Health Professions, University at Buffalo, Buffalo, NY, USA

## Abstract

**Background:**

Recommendations about precautionary behaviors are a key part of public health responses to infectious disease threats such as the 2009 H1N1 pandemic. Individuals' interpretation of recommendations, willingness to comply, and factors predicting willingness were examined.

**Methods:**

A telephone survey of adult residents of New York State was conducted (N = 807). Respondents reported how they interpreted recommendations, willingness to engage in recommended actions, risk perceptions for H1N1 infection, and perceived efficacy of recommendations. Demographic characteristics were used to calculate sampling weights to obtain population-representative estimates.

**Results:**

There was substantial variability in interpretation of preventive actions. Willingness to engage in preventive actions also varied substantially; vaccination willingness was substantially lower than other preventive actions. No pattern of demographic characteristics consistently predicted willingness. Perceived efficacy was associated with willingness for all recommendations, and perceived severity was associated with willingness for some recommendations.

**Conclusions:**

Results suggest that individual interpretation of actions differ widely. The results suggest that current recommendations are not clear to laypeople and are open to different interpretations. These varying interpretations should be considered in crafting public health messages about precautionary behaviors.

## Background

In April 2009 a novel strain of influenza, 2009 H1N1 Influenza (colloquially referred to as "swine flu"), emerged as a public health threat [1]; pandemic transmission of the 2009 H1N1 Influenza was declared by the World Health Organization in June 2009 [2]. In the first year following emergence, between 43 and 88 million cases occurred, resulting in between 8,000 and 18,000 deaths [3]. In response to the emergence of the 2009 H1N1 Influenza, the U.S. Centers for Disease Control and Prevention (CDC) issued recommended precautions for individuals to take to limit disease transmission [4,5]. Recommended actions included washing hands, covering mouth when coughing, avoiding contact with sick individuals, staying home if sick, and engaging in social distancing behaviors.

The effectiveness of influenza prevention precautions such as those issued by the CDC is dependent on a substantial portion of the population engaging in the recommended precautionary behaviors [6,7]. We know from examination of responses to other infectious disease recommendations that individuals' willingness to engage in preventive measures varies widely depending on the specific actions being recommended [8-10]. Early news reports about the 2009 H1N1 Influenza response indicated significant resistance to vaccination [11,12], and reports from public opinion surveys suggested low rates of vaccination [13,14]. Moreover, willingness to take part in many of the precautionary behaviors recommended by the CDC may be influenced by factors other than the health-related value of the precaution (e.g., not making contact such as shaking hands might be impacted by the normative pressure to engage in expected social behaviors; staying home from work may be influenced by economic pressures to earn income). Willingness to engage in precautionary behaviors may therefore vary both across specific precautions and across individuals depending on the other factors influencing behavior [15].

Given the importance of precautionary measures in preventing the emergence and progression of infectious disease pandemics, it may be helpful to explore the public's understanding of precautionary recommendations and their willingness to engage in them. This is especially true in the context of pandemics, where accurate comprehension and quick compliance with recommendations is necessary to halt the spread of the pathogen [6]. A misinterpreted recommendation can give the dangerous illusion of doing what needs to be done, when, in reality, pathogen transmission is not prevented. Although precautionary behaviors have been examined among health care providers [16-18] and members of specific high risk populations [19-22], less is known about the general public's response to such recommendations. Vaccination rates have been shown to be related to demographic characteristics [22,23], perceptions of risk for contracting illness [19,23,24], and beliefs about vaccine efficacy [25-27]. The extent to which these findings extend to precautionary behaviors involving more complex individual behaviors (e.g., altering social interaction patterns) is not clear; examination of early responses to H1N1 suggests that perception of risk and efficacy is related to behavioral uptake [8].

We examined several factors related to the willingness of the general public to engage in the CDC-recommended precautions to prevent 2009 H1N1 Influenza. First, we examined how individuals interpreted CDC's precautionary recommendations for 2009 H1N1 Influenza prevention. Second, we examined willingness to comply with the recommended precautionary measures. Third, we explored whether willingness to comply varied according to demographic and socioeconomic characteristics. Finally, we examined whether perceptions of risk for 2009 H1N1 Influenza infection and beliefs about the efficacy of precautionary behaviors related to willingness to comply.

## Methods

### Participants and Survey Methods

The data reported here were collected in a statewide opinion poll conducted by the Survey Research Center at Stony Brook University; the University's IRB approved the protocol for the opinion poll. Adult residents of New York State (N = 807) took part in the telephone survey. Participants were recruited via random digit dialing of telephone numbers in New York State; the sample of phone numbers included the entire state of New York, to allow for coverage of the entire state in the survey. Exclusionary criteria included non-English speaking households, as well as individuals physically or mentally incapable of completing the survey. When households were reached, the most recent birthday method was used to select an individual participant from the household (i.e., selecting the adult household member with the birthday closest to the date the survey was administered).

Participants were recruited and completed the study between October 14 and November 24, 2009. Of those households successfully contacted by an interviewer, there was a 24% cooperation rate [28]. Participant demographic characteristics (gender, age, race, educational attainment, and region of the state) were used to calculate sampling weights so that analyses reflect the state population as a whole (see *Analysis Strategy *below). Demographic characteristics of the sample (based on sampling weight corrected analyses) are presented in Table 1.

**Table 1:** Descriptive Characteristics of Participants

Characteristic	%
Gender	
Female	52
Male	48
	
Race/Ethnicity	
White	64
Black/African American	14
Hispanic/Latino	9
Asian	6
Native American or Alaskan Native	1
	
Age	
18-34	29
35-49	27
50-64	25
65+	17
	
Number of Children	
None	61
1 or more	37
	
Education	
< High school diploma	16
High School graduate	28
Some college, no degree	23
Bachelor's degree (BA, AB, BS)	18
> Bachelor's degree	13

Note: numbers presented are weighted using the calculated sampling weights (see *Methods*)

### Measures

A complete set of the key measures described below can be found in Additional File 1.

### Perceptions about Preventive Actions

Participants were asked a set of open-ended questions to assess their recognition of and understanding about preventive actions. First, prior to answering any closed-ended questions about specific precautions, participants were asked to list all of the recommended precautions they had heard about for 2009 H1N1 Influenza prevention. Next, participants were asked to report their interpretation of the instructions to "avoid close contact with sick people" and to "wash your hands often".

### Perceptions of Swine Flu Risk

Three components of risk perception were assessed: severity, likelihood, and degree of worry. For *perceived severity*, participants were asked: "If you were to get infected with the swine flu, how serious of a health issue would it be for you?" Responses were made on a 5 point scale ranging from not at all serious to extremely serious. *Perceived likelihood *was assessed with the item: "How likely are you to get infected with the swine flu this fall?" with 5 response options ranging from not at all likely to extremely likely. Participants reported *perceived worry *by responding to the item "How worried are you about getting infected with the swine flu this fall?" A five point response scale ranging from not at all worried to extremely worried was provided.

### Efficacy of and Willingness to Engage in Preventive Actions

Participants were asked about their beliefs concerning the efficacy of and their willingness to engage in various preventive actions recommended to prevent spread of 2009 H1N1 Influenza. The preventive actions were drawn from the Centers for Disease Control and Prevention precaution recommendations. Participants were asked about the following: covering your nose and mouth with a tissue when you cough or sneeze; washing your hands often with soap and water; cleaning your hands with an alcohol-based hand cleaner; avoiding touching your eyes, nose, or mouth; avoiding close contact with sick people; staying home if you are sick for seven days after symptoms begin; and getting a vaccination.

To assess perceived efficacy, for each preventive action participants responded to the question: "Is [covering your nose and mouth with a tissue when you cough or sneeze] effective at keeping you and others from getting the flu?" To measure willingness to engage in each preventive action, participants were asked: "Would you be willing to [cover your nose and mouth with a tissue when you cough or sneeze] to prevent the flu?" For both efficacy and willingness, a yes/no response option format was used.

### Demographics

Participants reported age, gender, race/ethnicity, household income, education, whether there were children living in the home at the time of the survey, and employment status.

### Analysis Strategy

Participant demographic characteristics were used to weight the sample based on gender, age, race, education, and region of the state. Based on population data from the 2006-2008 U.S. Census American Community Survey, weights were calculated in order to reflect the overall demographics of New York State, thus allowing for estimation of population representative results. All reported analyses were conducted in STATA 11 using survey analysis techniques to incorporate the sampling weights.

Responses to the open-ended questions were reviewed by all authors and set of inclusive categories describing the responses were developed. Each participant's open-ended questions were then coded into categories by one co-author (KS) and reviewed by a second co-author (MK). Descriptive statistics were used to summarize responses to the open-ended precaution questions. Descriptive statistics were used to summarize the proportion of participants who believed each preventive action to be efficacious and who were willing to engage in each preventive action; differences in the proportion across preventive actions was assessed using Newcombe's [29] method (method 10) for comparing within-participant paired proportions. To examine predictors of willingness to engage in each preventive action, logistic regression analyses were conducted in which willingness to engage in each preventive action (no, yes) was used as a categorical criterion variable and, for each analysis, demographics, perceived risk, perceived worry, and perceived efficacy were entered as predictor variables.

## Results

### Perceptions of Preventive Actions

Table 2 reports the percentage of respondents who self-generated various preventive actions (respondents were prompted to generate as many as preventive actions as they could, so percentages add up to greater than 100%) for 2009 H1N1 Influenza prevention. With the exception of handwashing, no preventive action was mentioned by more than 20% of respondents. Overall, 3% reported no actions, 21% reported one preventive action, 48% reported two actions, 20% reported three actions, and 8% reported four or more preventive actions.

**Table 2:** Reporting and Interpretation of Recommendations to Prevent 2009 H1N1 Influenza

Open-Ended Responses to Question about What Recommendations Were Made to Prevent Transmission	Sample Participant Responses	% of Respondents
Wash hands	"washing your hands frequently" "wash your hands all the time"	76
Avoid Sick People	"stay away from sick people" "avoid people with swine flu"	16
Cough/sneeze into sleeve	"coughing into elbow" "sneezing into sleeves"	16
Cover your mouth when coughing/sneezing	"cover your sneezes" "covering your cough"	16
Get vaccination	"get flu shot" "getting the flu vaccines"	16
Sanitize hands	"use purel when you get in the house" "use sanitizer"	16
Avoid crowds/enclosed places	"stay out of public places" "stay out of congested areas"	13
Stay home/rest	"staying home if you are sick" "stay home if you feel ill"	9
Good hygiene	"good hygiene" "keep things clean"	8
Good diet/eating well/exercise	"be careful what you eat" "keep yourself well (eating, exercise)"	6

There was great variability in the interpretation of the instruction to "wash regularly": 9% of respondents reported less than 5 times per day, 21% reported 5 or 6 times per day, 14% reported 7 or 8 times per day, 15% reported 9-10 times per day, and 24% reported more than 10 times per day; 13% gave a non-numeric response.

There was substantial variability in the interpretation of the instruction to "avoid sick people" (Table 3). The majority of responses dealt specifically with avoiding overall interactions with a specific person one knows to be sick (e.g., "not being close to someone who has the flu"). A much smaller subset of responses included other means of avoiding contact, such as general distancing from social situations and interactions (i.e., avoiding crowds to limit exposure to infected individuals) and avoiding direct physical contact (e.g., "don't shake their hands or kiss them"). Also of note is that over 10% of responses were unrelated to avoidance of infected individuals (e.g., using facemasks, engaging in good hand hygiene habits).

**Table 3:** Interpretation of Recommendation to "Avoid Sick People"

Interpretation of Recommendation	Sample Participant Responses	% of Respondents
Avoid Social Contact	"not going near people that are sick" "don't go see people who have swine flu" "not being close to someone who has the flu"	72
Social Distancing	"stay away from crowds" "stay away from doctors offices, hospitals, daycare" "avoiding children, nursing homes, hospitals, and friends/family"	12
Use a Face Mask	"take precautions like wearing a mask" "use a mask" "wear a mask"	4
Hand Hygiene Techniques	"going shopping wipe off shopping cart" "washing your hands" "wash your hands if you come in contact with sick people"	9
Avoid Physical Contact	"not kissing or hugging people" "not touching" "don't shake their hands or kiss them"	24
Extraneous	"10 foot pole" "just what it says" "not to catch the germs"	5

### Descriptive Statistics: Perceived Risk, Efficacy, and Willingness to Engage in Precautions

Table 4 reports the percentage of participants endorsing the efficacy of each precautionary behavior and the percentage reporting willingness to engage in each behavior. Regular hand washing was reported as efficacious by the highest number of participants (95%). With the exception of vaccination, all remaining precautions were believed to be efficacious by at least 88% of the respondents. Perceived efficacy of vaccination was significantly lower than that of any other precaution, with only 81% of respondents believing the vaccine to be effective at preventing swine flu infection.

**Table 4:** Perceived Effectiveness of and Willingness to Engage in Precautionary Behaviors to Prevent 2009 H1N1 Influenza

Behavior	Proportion indicating effectiveness of behavior (95%CI)	Proportion reporting willingness to engage in behavior (95%CI)
Covering Mouth to Cough	93.1% (90.7, 95.5)	98.4% (97.1, 99.7)
Hand washing	96.1% (94.1, 98.1)	98.5% (97.3, 99.9)
Using Hand Sanitizer	88.4% (85.2, 91.7)	88.8% (86.0, 91.5)
Avoid Touching Your Eyes	88.7% (85.4, 91.9)	90.3% (86.9, 93.7)
Avoid Close Contact	93.9% (91.3, 96.6)	93.2% (90.7, 95.9)
Staying Home	92.6% (89.6, 95.6)	87.7% (84.0, 91.5)
Getting a Vaccine	80.9% (76.6, 85.2)	63.9% (59.0, 68.7)

Numbers in cells indicate percentage of respondents indicating they believe a precautionary behavior IS effective/that they would be willing to engage in the behavior

Willingness to engage in precautions also varied greatly. Nearly all respondents were willing to cover the mouth to cough (98%) and wash hands (98%). Vaccination was again significantly lower than all other precautions, with only 64% of the sample reporting a willingness to be vaccinated to prevent spread of the illness. At least 87% of the respondents were willing to engage in each of the remaining precautions.

### Relation of Demographic Characteristics to Willingness to Engage in Each Precautionary Behavior

Table 5 presents the results of multivariate logistic regression analyses estimating the relation of each demographic characteristic to participants' willingness to engage in each precautionary behavior. Participant age was related to willingness to avoid touching eyes, avoid close contact, and stay at home, with older respondents more willing to engage in each precaution. Respondents who worked outside the home were more willing to wash hands. Respondents with higher education levels were more willing to wash hands and to avoid touching eyes.

**Table 5:** Association between Demographic Variables and Willingness to Engage in Precautionary Behaviors for Prevention of 2009 H1N1 Influenza

	Willing to Cover Mouth to Cough OR (95% CI)	Willing to Wash Hands OR (95% CI)	Willing to Use Hand Sanitizer OR (95% CI)	Willing to Avoid Touching Your Eyes OR (95% CI)	Willing to Avoid Close Contact OR (95% CI)	Willing to Stay Home OR (95% CI)	Willing to Get a Vaccine OR (95% CI)
Age (continuous)	1.04 (0.97, 1.11)	1.04 (0.98, 1.12)	1.01 (0.98, 1.04)	2.08* (1.00, 1.06)	1.04 ^** ^**(1.01, 1.08)**	1.03 ^* ^**(1.00, 1.06)**	1.01 (0.99, 1.03)
Child In Home (Reference = No)	3.43 (0.29, 40.31)	7.98 (0.81, 78.35)	1.03 (0.48, 2.21)	2.06 (0.83, 5.16)	1.25 (0.47, 3.30)	.81 (0.366, 1.82)	0.84 (0.51, 1.40)
Work outside of home (Reference = No)	6.89 (0.90, 52.77)	11.82* 1.69, 82.41)	1.12 (0.49, 2.60)	1.27 (0.54, 2.99)	2.11 (0.82, 5.42)	.71 (0.20, 2.54)	1.04 (0.58, 1.89)
Education (Ref = less than high school)							
High school graduate to less than college degree	2.52 (0.52,12.01)	7.05* (1.00, 50.08)	0.79 (0.24, 2.60)	1.61 (0.62, 4.16)	2.05 (0.67, 6.33)	0.37 (0.68, 1.99)	0.51 (0.23, 1.15)
Bachelors Degree or greater	3.15 (0.75, 13.28)	1.86 (0.51, 6.79)	0.57 (0.19, 1.71)	2.76* (1.06, 7.23)	1.51 (0.54, 4.26)	0.37 (0.07, 1.81)	0.64 (0.29, 1.42)
Race--White versus Other	0.69 (0.17, 2.82)	0.37 (0.08, 1.71)	1.87 (0.82, 4.22)	0.67 (0.29, 1.55)	1.37 (0.52, 3.67)	1.01 (0.49, 2.08)	1.12 (0.67, 1.86)

Notes: Numbers in cells are odds ratios; all analyses are adjusted using sampling weights (see *Methods)*

+ p-value < .1 but > .05

* p-value < .05 but > .01

** p-value < .01 but > .001

*** p-value < .001

### Relation of Severity, Likelihood, Worry, and Efficacy to Willingness to Engage in Each Precautionary Behavior

The multivariate results for relation of severity, likelihood, worry, and efficacy to willingness to engage in each precautionary behavior are presented in Table 6. As can be seen in the table, higher perceived severity was associated with greater willingness to use hand sanitizer, stay home, and get vaccinated. Perceived likelihood and worry were not significantly associated with any of the preventive actions. Perceived efficacy was significantly related to each preventive action; higher perceived efficacy was associated with greater willingness to engage in the preventive action.

**Table 6:** Association between Psychosocial Variables and Willingness to Engage in Precautionary Behaviors for Prevention of 2009 H1N1 Influenza

	Willing to Cover Mouth to Cough OR (95% CI)	Willing to Wash Hands OR (95% CI)	Willing to Use Hand Sanitizer OR (95% CI)	Willing to Avoid Touching Your Eyes OR (95% CI)	Willing to Avoid Close Contact OR (95% CI)	Willing to Stay Home OR (95% CI)	Willing to Get a Vaccine OR (95% CI)
Perceived Risk	0.99 (0.49, 2.01)	0.57 (0.28, 1.13)	1.07 (0.77, 1.48)	1.51+ (0.93, 2.45)	.80 (0.53, 1.19)	0.86 0.68, 1.10)	1.18 (0.89, 1.55)
Perceived Severity	1.50 (0.63, 3.53)	4.40+ (0.83, 23.29)	1.89*** (1.42, 2.52)	1.07 (0.65, 1.77)	0.99 (0.66, 1.49)	1.51* (1.06, 2.17)	1.44** (1.16, 1.79)
Perceived Worry	0.90 (0.29, 2.83)	1.02 (0.33, 3.21)	1.13 (0.80, 1.59)	0.59 (0.30, 1.17)	1.44+ (0.93, 2.23)	1.21 (0.83, 1.77)	1.24 (0.93, 1.66)
Perceived Efficacy	53.05*** (12.29, 228.89)	26.98*** (3.33, 218.68)	16.49*** (8.00, 33.97)	8.53*** (3.63, 20.01)	24.90*** (8.54, 72.56)	8.55*** (3.13, 23.37)	18.29*** (8.82, 37.94)

Notes: Numbers in cells are odds ratios; all analyses are adjusted using sampling weights (see *Methods)*

+ p-value < .1 but > .05

* p-value < .05 but > .01

** p-value < .01 but > .001

*** p-value < .001

## Discussion

Several important findings emerged from the results of this telephone survey conducted during the 2009 H1N1 Influenza pandemic. Unprompted knowledge about preventive actions recommended by CDC was relatively limited--only one of the preventive actions was spontaneously thought of by more than 20% of the respondents. This is especially surprising given that the survey was conducted more than 5 months after the CDC issued initial recommendations to prevent transmission. Moreover, participants had widely varying interpretations of broad, general recommendations issued by CDC--washing hands often could mean anything from 5 times a day to 20 times a day, depending on the participant, and avoiding sick people was interpreted as involving a variety of different specific behavioral actions across participants, ranging from avoiding social situations to using hand sanitizer.

Second, there were marked differences across specific precautionary behaviors in both perceptions of efficacy and willingness to engage in preventive actions. In particular, both perceived efficacy of vaccination and willingness to be vaccinated was substantially lower than any other preventive action. Although participants were substantially more willing to engage in other preventive actions, there was still a difference of more than 10% in willingness to engage in various other precautions.

Also of interest are the findings concerning predictors of willingness to engage in each behavior. No demographic or socioeconomic factor predicted willingness to engage in every precautionary behavior and there was no consistent profile of demographic predictors across behaviors. By contrast, psychosocial variables, in particular perceived precaution efficacy and severity of swine flu, were predictive of willingness to engage in preventive actions.

### Implications

The findings of the study have several implications for public health practice. Educational messages around infectious disease emergencies such as the H1N1 pandemic cannot be assumed to be clear to the public. The findings concerning respondents' interpretation of messages about preventive actions have implications for how messages are designed and presented. For example, the broad category message "avoid contact with sick people" was interpreted in a variety of different ways by respondents and there was little consistency in what specific actions individuals thought were suggested by the message. This suggests that communications about preventive actions might be more effective if they focused on specific, concrete behavioral strategies either instead of or in addition to global, abstract categories of behavior, similar to suggestions made for using concrete behavioral checklists to encourage behavior change in medical contexts [30].

Second, health communication strategies should consider differential targeting and differential investment of time and resources to messages about particular behavioral actions. The findings concerning perceived efficacy and willingness for different preventive actions show substantial differences across actions. Some preventive actions were accepted by close to all respondents (e.g., washing hands) whereas others were less widely accepted (e.g., staying home from work, vaccination). Knowledge about differential acceptability would allow for targeting resources to designing and implementing education strategies concerning those actions which are least accepted.

It is also important to consider why the acceptability of various preventive actions might differ. One factor may concern the amount of effort involved in engaging in the action and the other costs involved with the action. For example, both staying home from work and vaccination require more effort (e.g., scheduling an appointment and going to a health care provider) and have more associated costs (e.g., lost work time and lost income) than an action like covering one's mouth when coughing. A second factor may be emotional/visceral reactions to some of the behavioral actions [12,31]. For example, there may be fear associated with getting vaccinated and a feeling of disgust associated with using hand sanitizer. Locus of control might also differ across behaviors. For example, covering one's mouth when coughing may be perceived as more within the individual person's locus of control than an action like vaccination, which might be seen as controlled by a health care provider. In addition, given our findings about the relation between perceived efficacy and willingness to engage in preventive behaviors, considering the public's perceptions of efficacy of recommended actions is necessary and efforts to increase perceptions of efficacy should be considered.

Finally, the findings about the relation of risk perceptions to engagement in preventive actions suggest that health communications concerning precautions should not only address the precautionary behavior, but should also communicate the degree of risk associated with a given health problem. Given the variability in perceived risk across individuals and that degree of perceived risk predicts taking action, using health communication strategies to address inaccurate risk perceptions may make messages about precautionary behaviors more effective.

### Study Limitations

The survey was cross-sectional and, thus, the relations between variables (e.g., risk and demographics as predictors of willingness) are properly interpreted as associations between variables and not as causal. Second, given the time and space limitations of the telephone survey format, most constructs were assessed with single item measures. In addition, the questions asked about the role of behaviors in preventing spread of influenza without differentiating between transmission of the virus from the respondent to another person versus infection of the respondent by another person. Examining whether this distinction influences perceived efficacy and willingness to engage in behaviors would be a useful question for future research. Third, the timing of the survey was such that questions were asked several months after the start of the pandemic. Given the cross-sectional design, there was likely no one optimal time point for conducting a survey about a dynamic infectious disease process. However, the timing of the survey relative to the development of the pandemic should be considered in interpreting the results. The survey took place >5 months after the start of the pandemic and after numerous health education messages were in the field and, still, we found relatively low levels of knowledge of actions to prevent the 2009 H1N1 Influenza. The timing of the survey is also of interest when considering responses concerning vaccination, given the shifting availability of vaccinations for 2009 H1N1 over time. Finally, the behavior measure in the study is a self-report measure of willingness to engage in a given preventive behavior and is a categorical, yes/no response measure. The choice to use such a measure was purposeful given that some of the preventive behaviors are ones that individuals might not have had the opportunity to enact (e.g., a participant could only have covered the mouth when coughing if the participant had been sick). However, it is important to note that there is not a one-to-one correspondence between willingness to engage in a behavior and actual behavioral practices [32]. The results for the willingness measure are consistent with those of other reports of acceptability of vaccination using Likert-type response scales [13], increasing confidence in the accuracy of the results. However, the reported willingness rates in this study may be higher than actual engagement in behaviors. For example, actual uptake of influenza vaccination in New York State at the time of the survey was just under 20% [33].

## Conclusions

The results reported here indicate that individual interpretation of recommended preventive actions may not match the intended meaning. Interpretations can vary widely and can be inaccurate. In addition, there is great variability in acceptability of precautions. The nature of varying and incorrect interpretations of precaution recommendations must be considered when crafting public health messages recommending behavioral actions on the part of individuals.

## Competing interests

The authors declare that they have no competing interests.

## Authors' contributions

The study was conceived and measures developed by MTK, PKR, and LTK. Data analysis was conducted by MTK and KMS. The manuscript was written by MTK. All authors read, edited, and approved the final manuscript.

## Pre-publication history

The pre-publication history for this paper can be accessed here:

http://www.biomedcentral.com/1471-2458/11/152/prepub

## Supplementary Material

Additional file 1 **Questionnaire Items**. This file contains the questionnaire items used to assess key constructs reported in the paper. Click here for file
