# Supplementary material for: Perceptions of and willingness to engage in public health precautions to prevent 2009 H1N1 influenza transmission
Source: BMC Public Health. 2011 Mar 8;11:152. doi: 10.1186/1471-2458-11-152 (PMC3062607; doi:10.1186/1471-2458-11-152)
Supplement: Additional file 1 — Questionnaire Items. This file contains the questionnaire items used to assess key constructs reported in the paper. [file 1471-2458-11-152-S1.DOC]

Questions about H1N1 Precautionary Behaviors

Kiviniemi, Ram, Kozlowski, and Smith

BMC Public Health Paper

1) First, have you heard about the "SWINE FLU," also known as H1N1 influenza?

1. Yes

2. No

*If the answer is anything other than 1, skip to question 6*

2) How likely are you to get infected with the SWINE FLU this fall?

1. Not at all likely

2. Not likely

3. Somewhat likely

4. Very likely

5. Extremely likely

3) How worried are you about getting infected with the SWINE FLU this fall?

1. Not at all worried

2. Not worried

3. Somewhat worried

4. Very worried

5. Extremely worried

4) If you were to get infected with the SWINE FLU, how serious a health issue would it be for you?

1. Not at all serious

2. Not serious

3. Somewhat serious

4. Very serious

5. Extremely serious

5) Based on what you know or have heard about SWINE FLU, please tell me the symptoms of swine flu infection.

Can you think of any other symptoms of SWINE FLU infection?

[OPEN ENDED QUESTION]

6) A number of recommendations have been given about things you can do to make it less likely that you will get the flu. Please tell me which recommendations you have heard.

Can you think of any other recommendations you have heard?

I'm going to read you a list of some of the recommended flu prevention measures. Please tell me which ones you think are EFFECTIVE at keeping you and others from getting the flu.

*Respondents were asked 7-13 in random order.*

7) Is covering your nose and mouth with a tissue when you cough or sneeze effective at keeping you and others from getting the flu?

1. Yes

2. No

8) Is washing your hands often with soap and water effective at keeping you and others from getting the flu?

1. Yes

2. No

9) Is cleaning your hands often with an alcohol-based hand cleaner effective at keeping you and others from getting the flu?

1. Yes

2. No

10) Is avoiding touching your eyes, nose, or mouth effective at keeping you and others **from getting the flu?**

**1. Yes**

2. No

11) Is trying to avoid close contact with sick people effective at keeping you and others from getting the flu?

1. Yes

2. No

12) Is staying home if you are sick for 7 days after your symptoms begin or until you have been symptom-free for 24 hours, whichever is longer effective at keeping you and others from getting the flu?

1. Yes

2. No

13) Is getting a vaccination effective at keeping you and others from getting the flu?

1. Yes

2. No

I'm going to read you the same list of recommended flu prevention measures. Please tell me which ones you would be WILLING TO DO TO PREVENT THE FLU.

*Respondents were asked 14-20 in random order.*

14) Would you be willing to cover your nose and mouth with a tissue when you cough or sneeze to prevent the flu?

1. Yes

2. No

15) Would you be willing to wash your hands often with soap and water to prevent the flu?

1. Yes

2. No

16) Would you be willing to clean your hands often with an alcohol-based hand cleaner to prevent the flu?

1. Yes

2. No

17) Would you be willing to avoid touching your eyes, nose, or mouth to prevent the flu?

1. Yes

2. No

18) Would you be willing to try to avoid close contact with sick people to prevent the flu?

1. Yes

2. No

19) Would you be willing to stay home if you are sick for 7 days after your symptoms begin or until you have been symptom-free for 24 hours, whichever is longer, to prevent the flu?

1. Yes

2. No

20) Would you be willing to get a vaccination to prevent getting the flu?

1. Yes

2. No

21) When you hear the recommendation to "WASH YOUR HANDS OFTEN," how many times a day do you think that means?

[OPEN ENDED QUESTION]

22) When you hear the recommendation to "AVOID CLOSE CONTACT WITH SICK PEOPLE," what sorts of things do you think that refers to?

[OPEN ENDED QUESTION]

23) How many times a day do you currently wash your hands with soap and water?

[OPEN ENDED QUESTION]
